# A Case Report of Cerebral Autosomal Dominant Arteriopathy With Subcortical Infarcts and Leukoencephalopathy Misdiagnosed as Multiple Sclerosis

**DOI:** 10.7759/cureus.40986

**Published:** 2023-06-26

**Authors:** Kholoud Aljaberi, Amna Ahli, Sudhir Kumar Palat Chirakkara, Ahmed Shatila

**Affiliations:** 1 Neurology Department, Sheikh Shakhbout Medical City, Abu Dhabi, ARE; 2 College of Medicine and Health Sciences, Khalifa University, Abu Dhabi, ARE

**Keywords:** demyelinating disease, stroke, small vessel disease, multiple sclerosis, cadasil

## Abstract

Cerebral autosomal dominant arteriopathy with subcortical infarcts and leukoencephalopathy (CADASIL) is a rare genetic disorder due to a NOTCH 3 mutation on chromosome 19 resulting in a small vessel disease that may mimic many other neurological disorders like migraine, stroke, transient ischaemic attack (TIA), dementia and psychiatric illnesses. The disease is confirmed by genetic testing and other investigations like MRI and skin biopsy are also helpful. Here, we present a 43-year-old male with a confirmed CADASIL through genetic testing, who was initially diagnosed as having multiple sclerosis due to recurrent attacks of focal neurological deficits in the form of weakness and vertigo and other progressive features like mental slowing and difficulties in performing the usual tasks at work, He had a strong family history of neurological illnesses from his mother’s side that made us think of an alternative diagnosis.

## Introduction

CADASIL or cerebral autosomal dominant arteriopathy with subcortical infarcts and leukoencephalopathy syndrome is an inherited systemic non-amyloid and non-atherosclerotic vasculopathy characterized by white matter lesions and subcortical lacunar infarcts. It is the most common hereditary cause of adult-onset stroke and vascular dementia [1]. It results from mutations of the NOTCH3 gene located on chromosome 19p13.1 having an autosomal dominant pattern of inheritance [2]. The presentation is variable, including stroke or transient ischaemic attacks (TIAs; 43%), migraine (40%) depression (9%) cognitive impairment (6%), and epilepsy (2-10%) [3]. The exact prevalence is unknown. One of the studies conducted by Razvi et al., using the national register of CADASIL in Scotland, estimated the prevalence of genetically confirmed CADASIL to be 1.98 per 100,000 adults and a probable mutation prevalence of 4.14 per 100,000 [4]. In another study, the prevalence of cysteine-changing NOTCH3 variants was found to be one in 452, about 100-fold higher than expected based on CADASIL prevalence estimations of two to five in 100,000 [5]. The literature from MENA (Middle East and Africa) region is sparse [6].

## Case presentation

A 43-year-old, right-handed male presented with left-hand weakness of three months duration. He was diagnosed to have multiple sclerosis (MS) previously. His symptoms started two years earlier with an episode of sudden-onset vertigo unrelated to head and body movements and left leg heaviness, which lasted for one day. After several months, he experienced two episodes of dysarthria and tongue heaviness lasting for a few hours. Three months before his presentation to us, he started to experience left-hand weakness not affecting his work along with cognitive slowing in the form of difficulty in calculation.

On reviewing the previous MRI, the diagnosis of MS was questionable because of the location and shape of the white matter lesions. He was born of a non-consanguineous marriage. His only sibling, his sister, was diagnosed with MS at the age of 40. Her illness was progressive, not responding to MS treatment. His mother who died in her 50s had obsessive-compulsive disorder with psychotic features. His three maternal uncles died in their 50s, two due to myocardial infarction and one due to stroke. All his three children were healthy. He denied any family history of dementia or migraine.

On examination, he scored 25 in MMSE. He had intact cranial nerves, decreased power over the left upper limb (4/5), deep tendon reflexes +3 without clonus, positive Babinski, normal sensory exam, intentional tremor in the left hand, and normal regular and tandem gaits.

**Figure 1:**
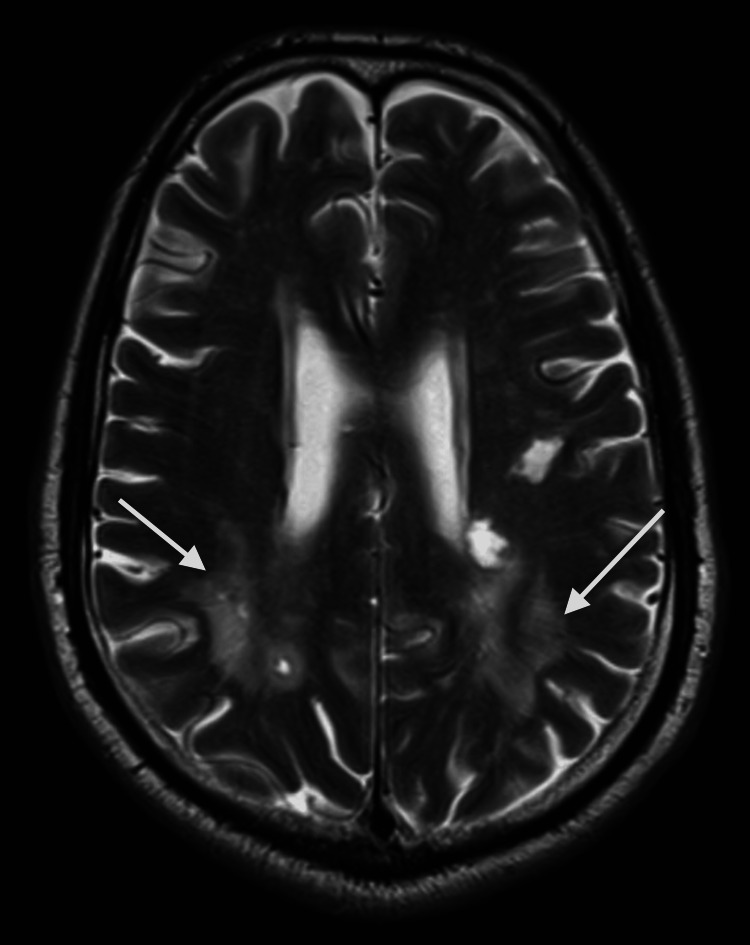
Brain MRI, T2, axial view

**Figure 2:**
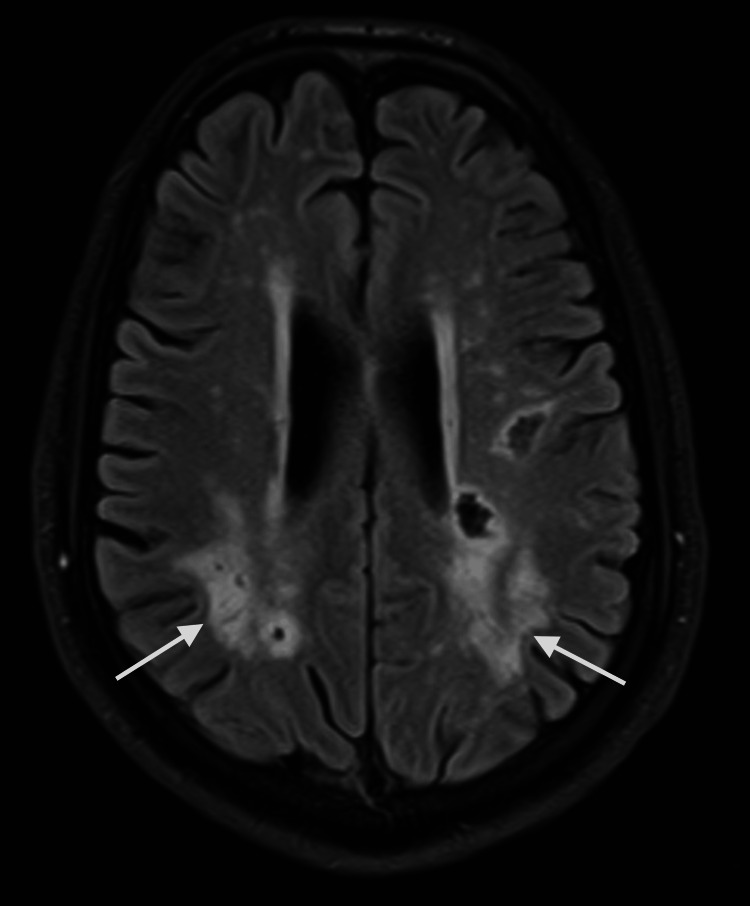
Brain MRI, FLAIR, axial view

**Figure 3:**
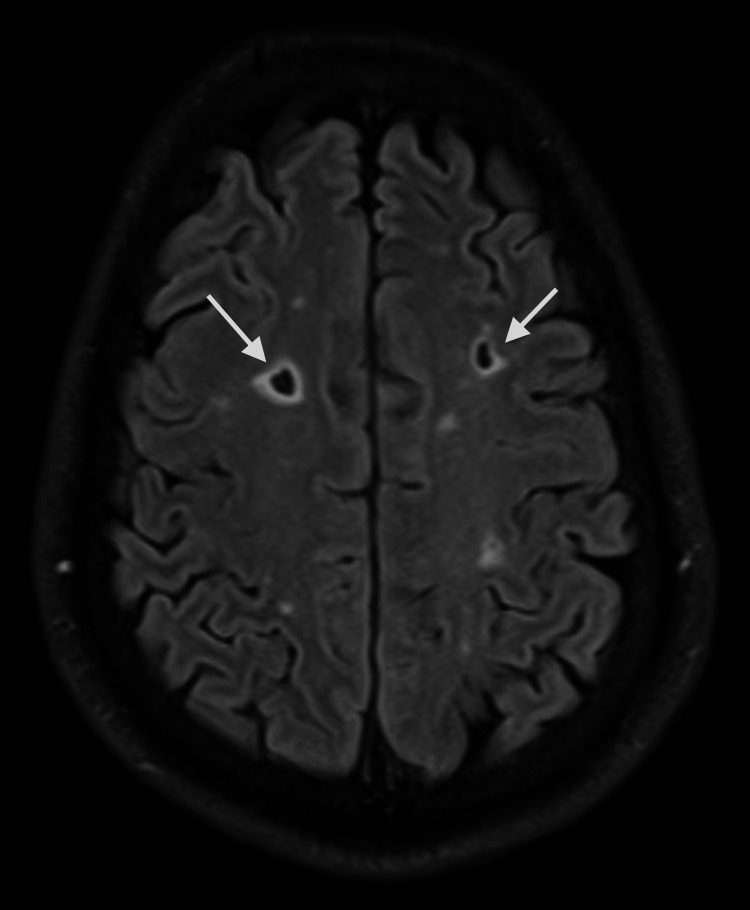
Brain MRI, FLAIR, axial view

**Figure 4:**
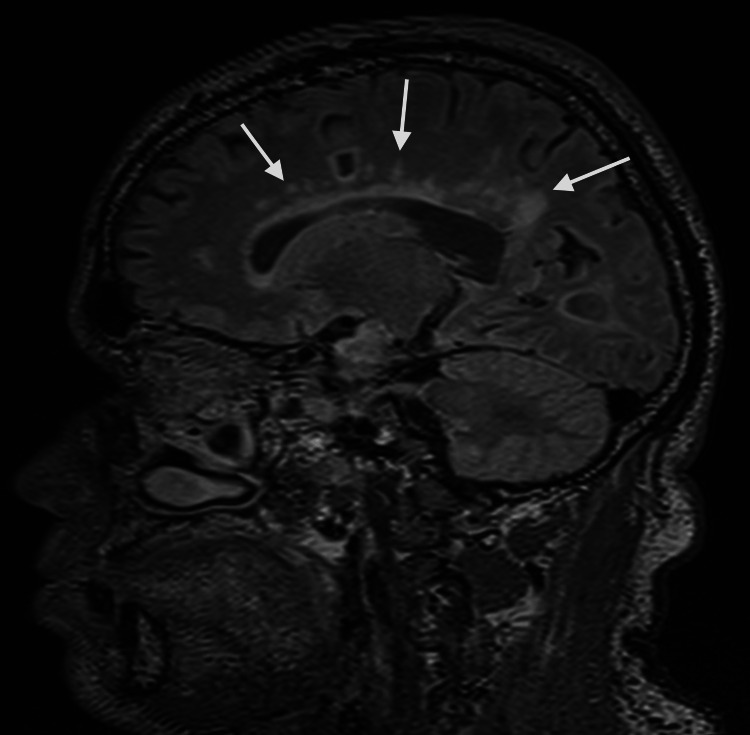
Brain MRI, FLAIR, sagittal view

MRI brain with and without contrast showed multiple white matter hyperintense lesions on T2 and fluid-attenuated inversion recovery (FLAIR) sequence in the frontoparietal region (Figures 1, 2). A few of them are irregular and show a central area of hypointensity (Figure 3). Symmetric confluent areas of white matter hyperintensity were seen in the peri-ventricular high parietal region extending from the subcortical to the periventricular region (Figure 4). No enhancing lesions were found following the administration of the contrast. CT angiography of the head and neck and echocardiogram were unremarkable. There were no oligoclonal bands in the cerebrospinal fluid. The autoimmune workup was negative.

Based on the extensive family history of neurological manifestations, we ordered a whole genome sequencing that was positive for a NOTCH3 mutation.

## Discussion

NOTCH3 mutations have been associated with CADASIL worldwide. The NOTCH3 gene is located on chromosome 19 [7]. It is a large gene that contains 33 exons encoding single-pass transmembrane receptors rich in epidermal growth factor repeats (EGFRs), found in the extracellular domain expressed on vascular smooth muscle wall and pericytes. It was described that there are over 200 mutations in NOTCH3. The protein has 2321 amino acids with 34 epidermal growth factor (EGF) contained in the extracellular domain (ECD) [8]. A total of 230 unique CADASIL mutations have already been reported [9]. Most cases are missense mutations involving cysteine amino acid. This resulted in an odd number of cysteine residues in a given EGF repeat [8]. In a normal EGF repeat, there are six cysteine residues forming three disulfide bonds. However, a mutation resulting in the formation of an odd number of cysteine residues disrupts the disulfide bonds causing structural abnormalities in the ECD. Further, the free cysteine residues undergo oligomerization and interfere with NOTCH trafficking, resulting in the clinical presentation of small vessel disease [7,8]. The function of NOTCH3 in vascular smooth muscle has been recently recognized. However, it is not yet clear whether CADASIL pathology occurs as indirect sequelae of abnormal NOTCH3 gene product accumulation or as a direct perturbed NOTCH signal regulation [4].

Clinical manifestations start between 20 and 40 years. Hypertension and smoking expedite ischemic events. Younger adults tend to present more with migraine with and without aura and ischemic events while older adults who are in their fifties experience recurrent ischemic events and cognitive decline. Dementia is seen after the age of 60, likely due to recurrent ischemic attacks. Other features like seizure, encephalopathy, and depression are also reported [10,11]. Life expectancy is reduced with an average age of death of 64.6 years in males and 70.7 in females [12].

CADASIL cases were misinterpreted previously until genetic testing became available. The clinical diagnosis is based on several conditions including (1) onset at age 40-50 years, (2) absence of stroke risk factors, (3) frequent lacunar infracts leading to pseudobulbar paralysis and dementia, and (4) family history of the disease suggesting autosomal dominant inheritance [13]. MRI abnormalities observed in CADASIL are signs of leukoaraiosis that may include multiple subcortical infracts, the presence of hyperintense signals in the white matter, cerebral microbleeds [14], enlarged perivascular spaces, and brain atrophy, and they tend to affect the temporal lobes and external capsule [9]. It is estimated that some MRI abnormalities can be seen and detected approximately 15 years prior to the onset of the disease [14]. Genetic testing is the gold standard to confirm CADASIL [13]. Histologically, it is characterized by the presence of a granular osmiophilic material layer around the vascular smooth muscles in the brain on electron microscopy. It may also be found in the skin, and hence biopsy can be used as a diagnostic modality for the disease [13].

CADASIL management is challenging, as there is no definitive therapy. Vascular risk factor management minimizes the chance of recurrent ischemic events. In acute ischemic events, thrombolysis is not recommended for deep ischemic events although it may be considered in case of a large vessel disease [15]. For migraine presentation, acetazolamide can be used as a prophylaxis, as it was found to increase cerebral blood flow [12]. For dementia, donepezil used in a multicenter trial showed some improvement in executive function [12].

## Conclusions

We discussed a middle-aged male who presented with recurrent episodes of neurological symptoms and signs with brain MRI features suggestive of white matter disease. He was initially misdiagnosed with multiple sclerosis. However, the strong family history and genetic tests confirmed CADASIL. CADASIL is a genetic disease that has variable clinical features, with stroke and migraine being the most common forms of presentation. The diagnosis was confirmed by genetic testing that revealed a NOTCH3 mutation, MRI played an important role in characterizing the lesions. Management was mainly supportive by addressing vascular risk factors for stroke and symptomatic treatment for the other associated symptoms.
